# Validation and Application of Quantitative Methods for D-Lactic Acid and L-Lactic Acid Determination in Lactic Acid Bacteria

**DOI:** 10.3390/foods15091537

**Published:** 2026-04-29

**Authors:** Chong Liu, Yiru Liu, Xuejian Yu, Yu Jing, Lu Zhang, Zhe Zhang, Lina Liu, Hairong Hu, Su Yao

**Affiliations:** 1 China National Research Institute of Food and Fermentation Industries, Beijing 100015, China; liuchong@china-cicc.cn (C.L.); rita@china-cicc.org (Y.L.); yuxuejian@china-cicc.org (X.Y.); jingyu@china-cicc.org (Y.J.); zhanglu@china-cicc.org (L.Z.); zhangzhe@china-cicc.org (Z.Z.); liulina@china-cicc.cn (L.L.); huhairong@china-cicc.cn (H.H.); 2 China Center of Industrial Culture Collection, Beijing 100015, China; 3 Beijing Advanced Innovation Center for Food Nutrition and Human Health, Department of Nutrition and Health, China Agricultural University, Beijing 100193, China

**Keywords:** probiotics, safety assessment, high-performance liquid chromatography, method validation, measurement uncertainty

## Abstract

The evaluation of D- and L-lactic acid production by lactic acid bacteria is of critical importance, particularly for strains intended for use in infant and young child foods. Additionally, compliance with relevant regulatory standards necessitates the detection of D-lactic acid. Additionally, regulatory requirements exist in this regard. This study aimed to develop and validate a method for simultaneously measuring D-lactic acid and L-lactic acid produced by lactic acid bacteria. The method validation of the HPLC analysis was performed in terms of accuracy, precision, specificity, limit of quantification (LOQ), linearity, range, and robustness, and the measurement uncertainty was also evaluated. The method demonstrated a limit of detection (LOD) of 0.25 µg/mL and LOQ of 0.8 μg/mL for D-/L-lactic acid. For six validated bacterial strains, mean recoveries ranged from 93.50% to 105.37%, with intra-assay relative standard deviations (RSD) of 0.90–2.64% and inter-laboratory RSD of 2.56–10.16%. Excellent linearity, accuracy, and precision were observed across the concentration range of 0.8–200.0 μg/mL. Results confirmed no interference from culture media batch variations, and sample stability was maintained for 48 h. Additionally, relative expanded uncertainties were determined as 10.48% and 7.64%. The developed method was suitable for the identification and quantification of D- and L-lactic acid in lactic acid bacteria fermentation broth samples. This method was applicable for assessing the production of D-/L-lactic acid by food cultures.

## 1. Introduction

Lactic acid is a crucial metabolic product in living organisms. Due to the chiral carbon atom at the α-position of the carboxyl group in its molecular backbone, lactic acid exists as two enantiomers: L-lactic acid and D-lactic acid. Lactic acid bacteria (LAB) are capable of producing D/L-lactic acid as the primary end product of carbohydrate fermentation, playing essential roles in food fermentation, probiotic function, and industrial biotechnology. For example, D-lactate serves as an essential precursor for the synthesis of heat-resistant poly-lactic acid (PLA), a biodegradable polymer widely used. The strains that produce high levels of D-lactic acid have great economic significance [1].

In humans, L-lactate is the predominant isomer and plays a central role in cellular energy metabolism. It is produced endogenously via anaerobic glycolysis, where pyruvate is reduced to L-lactate by L-lactate dehydrogenase (L-LDH) [2]. In contrast, D-lactate is produced in only trace amounts in human tissues and is primarily produced by gut microbial metabolism. Malabsorbed carbohydrates (CHO) could undergo fermentation in the colon, yielding D-/L-lactic acid. Colonic bacteria such as *Lactobacillus acidophilus*, *Lactobacillus fermentum*, *Lactobacillus delbrueckii* subsp. *lactis*, *Lactobacillus buchneri*, and *Streptococcus bovis* can synthesize D-lactic acid [3]. Exogenous sources include the consumption of foods like yogurt, apples, tomatoes, pickles, and beer, as well as certain administered pharmaceuticals [4]. A minor endogenous source of D-lactic acid arises from the methylglyoxal pathway [5].

The content of L-lactic acid is usually in a dynamic equilibrium process [6]; the metabolism of D-lactic acid is more noteworthy, although it accounts for approximately 1–5% of the serum lactic acid. The functional roles and metabolic pathways of D-lactate dehydrogenases (D-LDHs) in humans remain incompletely characterized [7]. Current evidence indicates that D-lactic acid degradation is catalyzed by the mitochondrial enzyme D-2-hydroxyacid dehydrogenase (D-2-HDH). This enzyme, which exhibits high activity in both the liver and renal cortex, metabolizes D-lactic acid at a rate approximately 20% of that achieved by L-LDH for L-lactic acid [8]. At the same time, the role of urinary excretion in patients with D-lactic acidosis will also be greatly reduced [9]. When excessive D-lactic acid is produced and accumulates, it can enter the bloodstream from the colon via monocarboxylate transporters (MCT-1 to MCT-8), be absorbed systemically, and transported into various cells, tissues, and organs [10]. When the rate of D-lactate production exceeds the body’s limited capacity for clearance, D-lactate accumulates in the bloodstream, leading to acidosis, along with neurological symptoms, including confusion, lethargy, memory loss, headache, and upper limb motor dysfunction [5]. It should be noted that the accumulation of D-lactate to clinically significant levels occurs primarily under pathological conditions involving the gastrointestinal tract; the threshold for toxic manifestations upon exceeding normal D-lactic acid concentrations exhibits individual variability [9,11].

Studies have shown that alterations in the gut microbiota may influence the accumulation of D-lactate [12]. For example, neurological symptoms like brain fog have been correlated with elevated D-lactate levels and gut bacterial overgrowth [13]. Additionally, recurrent D-lactic acidosis has been documented in pediatric patients with short bowel syndrome following the administration of probiotics containing *Lactobacillus acidophilus* and *Bifidobacterium infantis* [14]. Consequently, detecting and evaluating D-lactic acid production by food cultures, probiotics, particularly those intended for infant foods, is critically important. It should comply with the requirements of the relevant regulations and standards. The FAO/WHO guidelines [15] recommend assessing the D-lactic acid-producing metabolic activity of probiotic strains, even those on the list of Generally Recognized as Safe (GRAS). International standards such as CXS 72-1981 and CXS 74-1981 [16,17] stipulate that only L-lactic acid-producing cultures may be used, requiring demonstration of safety and appropriateness for these vulnerable populations. China’s national standard GB 31615.2 [18] mandates that for strains directly added to foods intended for infants under one year of age, D-lactic acid testing must be conducted using the inspection methods specified in national standards or other equivalent methods.

Currently, for the purpose of strain evaluation, a more stable and reliable analytical approach is desirable. Among methods for detecting D- and L-lactic acid content, high-performance liquid chromatography (HPLC) is the most extensively employed. This technique utilizes chiral stationary phases or chiral mobile phases to achieve enantiomeric separation of D- and L-lactic acid in samples, enabling simultaneous qualitative and quantitative analysis with a broad detection range, high sensitivity, excellent accuracy, and superior repeatability [19], making it a suitable candidate for providing a robust and standardized protocol for strain evaluation.

This study describes the development and validation of a chiral HPLC method for the quantitative determination of D- and L-lactic acid in LAB fermentation broths, aiming to provide a reliable and readily standardizable tool for inter-laboratory strain evaluation. Representative strains producing varying levels and ratios of D- and L-lactic acid were collected to validate the developed method. Furthermore, diverse LAB strains were selected to assess method applicability. This work aims to establish a technical reference for evaluating D- and L-lactic acid production by LAB, holding significant implications for the broader application of food cultures.

## 2. Materials and Methods

### 2.1. Reagents and Culture Media

CuSO_4_·5H_2_O and L-Cysteine monohydrochloride, all AR-grade, were supplied by Sigma-Aldrich (St. Louis, MO, USA). Additionally, MRS broth media from Beijing Land Bridge Technology Co., Ltd. (Beijing, China) were used.

### 2.2. Standards and Solutions

L-Lactic acid (CAS 79-33-4) and D-lactic acid sodium (CAS 920-49-0) high-purity analytical standards (≥98%) were purchased from Sigma-Aldrich (St. Louis, MO, USA); Stock solutions were prepared in ultrapure water at concentrations of 4.0 mg/mL for L-lactic acid and 5.0 mg/mL for sodium D-lactate. Mixed intermediate solutions (800 µg/mL of L-Lactic acid and D-lactic monomer) were prepared in a 10 mL glass volumetric flask by diluting these solutions in ultrapure water. Finally, mixed working standard solutions containing 1.0 to 200.0 µg/mL of D-/L-lactic were prepared in 10 mL glass volumetric flasks by diluting the mixed intermediate solutions with water. Stock and intermediate solutions were stored in the freezer at −20 °C. Working standard solutions were prepared before use.

### 2.3. Strains Selection and Collection

Representative strains were selected from the “List of Strains Permitted for Use in Foods for Infants and Young Children” and the “List of Strains Permitted for Use in Foods”, as issued by the National Health Commission of China [20]. Among these, 15 strains from the former list were isolated from commercial products. The remaining 23 strains were obtained from the China Center of Industrial Culture Collection (CICC). Detailed information was provided in Supplementary Table S1.

### 2.4. Strain Culture and Sample Preparation

The de Man–Rogosa–Sharpe (MRS) medium, widely used for the cultivation of LAB, was selected and supplemented with the reducing agent cysteine hydrochloride to improve anaerobic growth conditions and enhance strain viability [21]. LAB strains were transferred into modified MRS broth containing 0.05% L-cysteine hydrochloride (L-MRS) and cultured at 37 °C until the logarithmic growth phase, and subcultured to the second generation. Anaerobic strains were cultured under anaerobic conditions. Second-generation cultures were inoculated at 2% (*v*/*v*) into L-MRS liquid medium and incubated for 8, 12, 24, 36, 48, and 60 h. The obtained fermentation broth was centrifuged at 8000 r/min for 2 min, and the supernatant was collected. The supernatant was diluted 100 times with water and filtered through 0.22 μm PES syringe filters before being placed in a 2 mL glass vial for subsequent HPLC analysis. The uninoculated L-MRS medium was tested as a blank sample. To assess the statistical significance of the results obtained at different time points, one-way analysis of variance (ANOVA) followed by post hoc multiple comparisons was performed (*p* < 0.05).

### 2.5. Chromatographic Conditions

Chromatographic analysis was performed using an HPLC system equipped with a photo-diode array detector (PDA; Ultimate; Thermo Fisher Scientific, Waltham, MA, USA). Stereospecific assay of D- and L-lactic acid was achieved on a chiral column (MCI GEL CRS10W, 3 µm, 50 mm × 4.6 mm; Mitsubishi Chemical Group, Tokyo, Japan) coated with N,N-dioctyl-l-alanine as the chiral selector. To optimize the separation, various mobile phase compositions (1.2, 1.5, and 2.0 mmol/L aqueous copper sulfate solutions) and column temperatures (25 °C and 35 °C) were evaluated using a working standard solution of D-/L-lactic acid and a blank sample. Mobile phase was pumped at 0.5 mL/min, and UV detection was at 254 nm.

### 2.6. Calibration Curve and Sample Analysis

The working standard solutions of L/D-lactic acid prepared in Section 2.2 were analyzed at seven concentration levels, with triplicate measurements performed for each concentration. Calibration curves were constructed by plotting the response ratio against the corresponding concentration ratio. Heteroscedasticity was evaluated using two approaches: (i) an F-test at the 99% confidence level comparing the variances of the lowest and highest concentration levels (with n-1 degrees of freedom), and (ii) graphical analysis of the relative residual scatter plots. Based on the obtained results, an appropriate regression method—either ordinary least squares (OLS) or weighted least squares (WLS)—was selected to fit the optimal linear relationship for each analyte. The slope, intercept, and coefficient of determination (R^2^) were subsequently derived [22].

For quantifying, the contents of D-/L-lactic acid in the sample can be expressed as

(1) X = C_1_ × N_1_ − C_2_ × N_2_

where C_1_ was the amount of D-/L-lactic acid in a sample, C_2_ was the amount of D-/L-lactic acid in the blank sample, and N was the corresponding dilution factor.

### 2.7. Method Validation

Per the USP <1225> and ChP <9101> validation of compendial procedures, a full validation was performed for the quantification of the target analytes [23,24]. The performance characteristics considered for validation of the optimized method were: accuracy, precision, specificity, LOQ, linearity, range, robustness, and method uncertainty. Six strains were selected as validation strains, as shown in Table 1.

#### 2.7.1. Accuracy Evaluation

The accuracy of an analytical procedure is the closeness of test results obtained by that procedure to the true value. A spiking experiment was set up for the estimation and evaluation of the recovery. The fermentation broth samples were fortified with D- and L-lactic acid standard solution to obtain fortified samples containing five different expected concentrations. Three replicates per spiking level were prepared and analyzed, as well as three replicates of an unfortified sample. The results were expressed as recovery.

#### 2.7.2. Precision Evaluation

The precision of an analytical procedure is the degree of agreement among individual test results when the procedure is applied repeatedly to multiple samplings of a homogeneous sample. The precision of analytical procedure was expressed as the standard deviation (SD) and relative standard deviation (RSD) of measurements, including the evaluation of reproducibility and repeatability. Repeatability of the method was evaluated through ten repeated experiments on the same sample. Each strain was independently cultivated, processed, and analyzed following the complete sample preparation procedure.

Reproducibility of the method is assessed in an interlaboratory exercise. Six laboratories in China participated in the evaluation of the method for the quantification of D-/L-lactic acid produced by LAB. Each participating laboratory independently performed the entire workflow, including culture of the strain, sample processing, and HPLC analysis, conducted three independent measurements on each sample and the control sample. All participating laboratories were provided with a detailed Standard Operating Procedure (SOP) to ensure consistency in culture conditions and analytical parameters, particularly regarding the column and analysis conditions.

#### 2.7.3. Specificity Evaluation

A high degree of specificity is achieved by using chiral chromatographic columns for the detection of chiral molecules, and the consistency of retention times, and the chromatogram can be clearly displayed. No further assessment of specificity was done.

#### 2.7.4. Limit of Quantification (LOQ)

LOQ is the lowest amount of analyte in a sample that can be quantified with acceptable precision and accuracy. A signal-to-noise ratio of 10:1 was considered the acceptable LOQ, and determine whether this low concentration met the verification requirements by conducting a spiked experiment in the blank sample.

#### 2.7.5. Linearity and Range

Linearity refers to the ability of the method to obtain test results that are directly proportional to the concentration of the analyte in the sample within a given range. The range is between the upper and lower levels of analyte that have been demonstrated to be determined with a suitable level of precision, accuracy, and linearity using the procedure. In this experiment, linearity was established using six concentration levels by performing spike recovery tests in blank samples, in accordance with the ICH harmonized guideline [25]. The matrix effect was evaluated by comparing the slope ratio (% ME) of the matrix-matched calibration curve (m_M_) to the direct calibration curve (m_S_) [26].

#### 2.7.6. Robustness Evaluation

The robustness of the method is a measure of its capacity to remain unaffected by small but deliberate variations in method parameters and provides an indication of its reliability during normal usage. This method involves culture and HPLC determination. Therefore, different batches of culture media and different sample storage times were selected for evaluation, and the RSD of the test results was expressed.

#### 2.7.7. Method Uncertainty Evaluation

**Figure 1:**
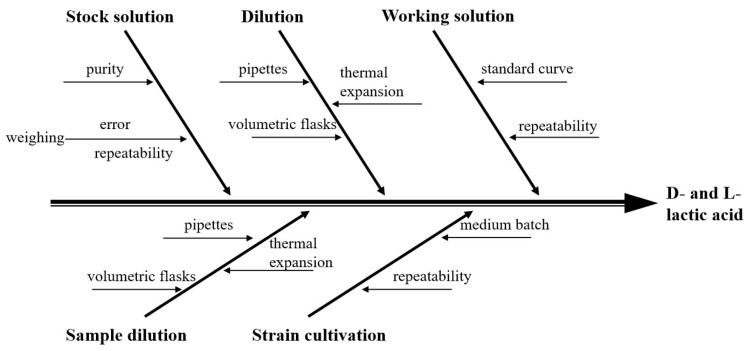
Cause-and-effect diagram for uncertainty.

The measurement uncertainty for D-/L-lactic acid produced by the strain is estimated according to ISO/IEC guide 98-3 [27]. Based on the analysis of the detection process and the mathematical model (Equation (1)), the main uncertainty sources were presented in the cause-and-effect diagram in Figure 1. These included standard purity, gravimetric preparation and dilution steps, as well as standard curve fitting; For the strain samples, comprising gravimetric preparation and dilution procedures, strain cultivation, and the uncertainty caused by the influence of the matrix detection during analysis. The relative combined uncertainty (U_rel_) was calculated with Equation (2). Since the laboratory has a temperature-controlled (air-conditioned) environment, the uncertainty from the temperature was neglected. For the assumption of distribution types for uncertainty components, the rectangular distribution was adopted for electronic balances, standards, etc., when only the limits of maximum permissible error are known without specific distribution information within the interval. For precision instruments such as volumetric flasks and pipettes, the triangular distribution was used because the actual volume during effective production is closer to the nominal value, and the probability of extreme values was low [28]. The uncertainty calculation method for the calibration curve obtained by the WLS regression method is shown in Table S4. The relative expanded uncertainty (U) values were found by multiplying the relative standard uncertainty by the coverage factor (κ = 2) for a 95% confidence level [29].

(2) $$U_{\mathrm{rel}}=\sqrt{U_{\mathrm{rel}}^{2}(P)\;+\;U_{\mathrm{rel}}^{2}(M)\;+\;U_{\mathrm{rel}}^{2}(V)\;+\;U_{\mathrm{rel}}^{2}(C)\;+\;U_{\mathrm{rel}}^{2}(\mathrm{Vs})\;+\;U_{\mathrm{rel}}^{2}(R)\;+\;U_{\mathrm{rel}}^{2}(\mathrm{CM})\;+\;U_{\mathrm{rel}}^{2}(\mathrm{am})}$$

## 3. Results

### 3.1. Method Development and Parameter Optimization

**Figure 2:**
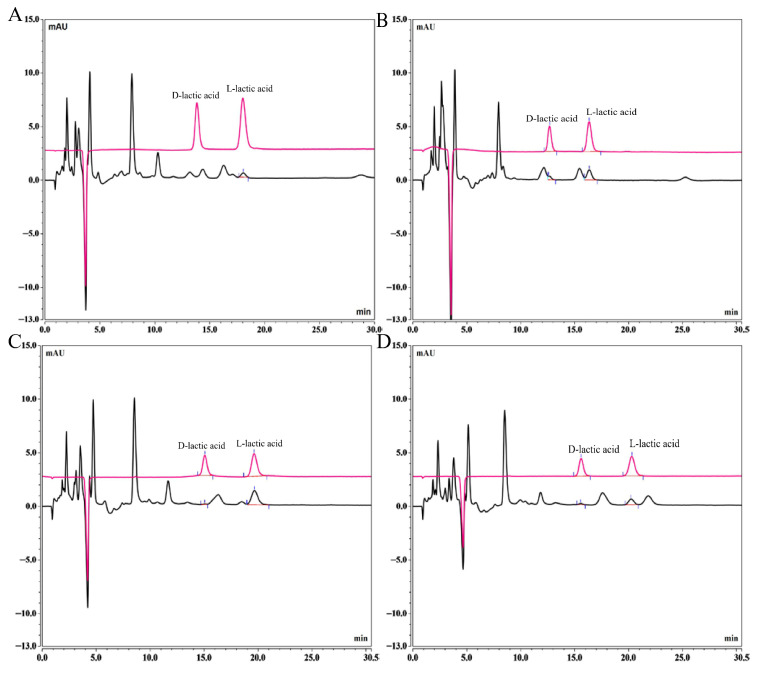
Optimization process of HPLC. (**A**) Mobile phase: 2.0 mM CuSO_4_·5H_2_O aq., column temperature: 25 °C, (**B**) 35 °C; (**C**) Column temperature 25 °C, mobile phase: 1.5 mM CuSO_4_·5H_2_O aq., (**D**) 1.2 mM CuSO_4_·5H_2_O aq.

Chromatographic condition optimization results are presented in Figure 2. Retention times were used to qualitatively determine the separation of D-/L-lactic acid peaks from impurity peaks. Under constant parameters (flow rate: 0.5 mL/min; detection wavelength: 254 nm; and injection volume: 10 µL), when using 2.0 mmol/L CuSO_4_·5H_2_O aqueous solution as mobile phase, baseline separation of D-lactic acid from adjacent impurities in culture medium samples was not achieved at column temperatures of 25 °C (Figure 2A) and 35 °C (Figure 2B). Higher temperatures progressively decreased resolution. At 25 °C with 1.5 mmol/L CuSO_4_·5H_2_O mobile phase, near-baseline separation of D-/L-lactic acid peaks was attained (Figure 2C). Complete separation of D-/L-lactic acid peaks from impurities was achieved using 1.2 mmol/L CuSO_4_·5H_2_O mobile phase at 25 °C (Figure 2D). By progressively diluting the D-/L-lactic acid mixed standard solution and combining it with the signal-to-noise ratio, the LOD of D-/L-lactic acid in this method was determined to be 0.25 µg/mL.

Heteroscedasticity in the response variances was assessed via an F-test and relative residual scatter plots. As all calculated F-values were greater than the critical values, and the residual plots displayed a distinctly non-random distribution, particularly evident at low concentrations (Table S2 and Figure S1), the weighted least squares (WLS) method was determined to be the optimal regression approach. Several weighting factors were evaluated, including 1/X^−2^, 1/X^−1^, 1/X^1^, and 1/X^2^. For each weighting scheme, the WLS model was fitted, and the log-likelihood value was calculated. The optimal weighting factor was selected as the one that maximized the log-likelihood, indicating the best correction for heteroscedasticity and the most adequate fit to the data. Consequently, a weight of 1/X^2^ was adopted for all subsequent calibrations.

**Figure 3:**
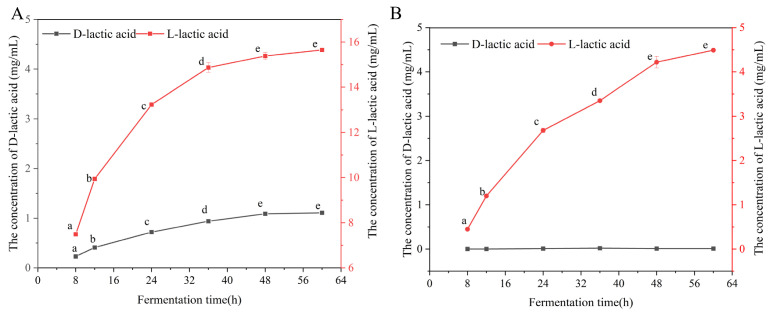
The concentration of D- and L-lactic acid produced by the strain under different fermentation times. CICC 10063R (**A**) and CICC 10036R (**B**).

The production of D-/L-lactic acid by *L. rhamnosus* CICC 10063R and *B. animalis* subsp. *lactis* CICC 10036R in L-MRS medium was monitored over different fermentation timepoints (Figure 3). For CICC 10063R, D- and L-lactic acid accumulation reached a plateau after 36 h. For CICC 10036R, D-lactic acid content remained below the LOD throughout fermentation, while L-lactic acid production plateaued after 48 h. Therefore, a standardized 48 h fermentation period in L-MRS medium was adopted for the quantification of D-and L-lactic acid in all subsequent analyses of LAB strains.

### 3.2. Performance Validation of the Established HPLC Method

The performance characteristics as specified were determined, and the method was successfully validated. The results of the various experiments are described below.

#### 3.2.1. Method Accuracy

Accuracy was evaluated in ninety determinations (five concentration levels by triplicate for each strain), and the results were within the acceptable range as suggested by the AOAC guidelines of validation [30]. For low concentrations, the results range from 70% and 120%, for high concentrations, the results range from 85% and 110%. The method accuracy varied between 95.27% and 104.50% at the D-lactic acid, 93.50% and 105.37% at the L-lactic acid; an overview of the recoveries and spiked concentration for all analytes is given in Table 2.

#### 3.2.2. Method Precision

Table 3 presents the precision of the analytical method. Repeatability, expressed as the RSD of ten biological replicate measurements, ranged from 0.90% to 2.64% depending on the analyte. Reproducibility was evaluated by analyzing results from six laboratories using three selected strains, CICC 10063R, CICC 10009R and CICC 10068R. The RSD for D-lactic acid detection were 10.16% and 7.39%. For L-lactic acid, all RSD values were all below 8%, which is compliant with the AOAC guidelines of validation [30]; RSD should be less than 8% for analytes at concentrations below 0.01%, and less than 11% at 10 μg/g.

#### 3.2.3. Specificity

**Figure 4:**
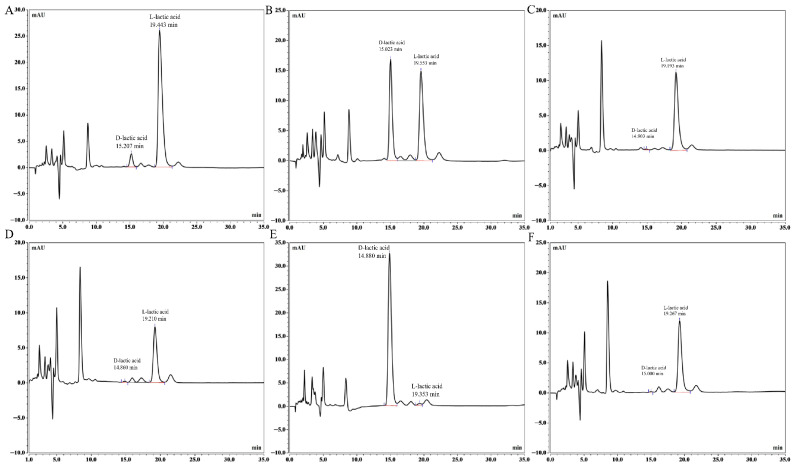
Chromatograms of validation strains: CICC 10063R (**A**), CICC 10009R (**B**), CICC 10068R (**C**), CICC 10036R (**D**), CICC 6047 (**E**), and CICC 6069 (**F**).

Using the established method for detection, the chromatograms of each strain are shown in Figure 4. Complete separation of D- and L-lactic acid was achieved, with baseline separation from impurity peaks. The peak purity match value for each sample was greater than 950, confirming the absence of co-eluting interferences; the results are presented in Table S5. Furthermore, the precision data demonstrate that the impurity peaks have no significant impact on the detection results of the two components.

#### 3.2.4. LOQ, Linearity and Range

The LOQ, estimated based on baseline noise analysis, was approximately 0.8 μg/mL. The uninoculated culture medium contains a small amount of D-and L-lactic acid, with slight variations in content between different batches. Based on this, adding 0.80/0.83 μg/mL of D-/L-lactic acid reference standard resulted in recovery rates and RSD that met the requirements for accuracy and precision across 10 tests (Table 4). Therefore, 0.8 μg/mL was established as the LOQ for this method, at which the analytes could be accurately quantified.

Similarly, different concentrations of reference standards were added to the uninoculated culture medium for validation through 10 experiments. The D-/L-lactic acid concentrations were 8.15/10.43, 32.59/41.70, 81.47/104.25, 154.79/156.38, and 194.75/198.08 μg/mL, respectively. The results were presented in Table S3, with average recovery ranging from 93.67% to 102.43% across all concentration levels, and repeatability RSD between 0.39% and 2.85%. In the linear regression equation of the matrix-matched calibration curve, the R^2^ > 0.9999, indicating excellent linearity. As shown in Table S3, the % ME values were 100.09% and 102.235, respectively; no significant matrix effect was observed [22]. Therefore, the detection results were accurate and reliable within the range of 0.8–200.0 μg/mL.

#### 3.2.5. Robustness

The method showed robustness since minor changes in the culture medium batch and samples were placed for 48 h after preparation. The test results of each strain are shown in Table 5. Under the two factors, the RSD of D-lactic acid and L-lactic acid contents were within the Repeatability (RSDr) requirements in AOAC.

#### 3.2.6. Method Uncertainty

The uncertainty of the quantification of D-/L-lactic acid produced by LAB was estimated using the HPLC method. Seven separate sources of uncertainty were considered in this study: U_rel_(P), the uncertainty associated with standard purity. The purities of D-lactic acid and L-lactic acid standards were 99% and 98%, respectively, with no uncertainty specified. Assuming a rectangular distribution (confidence factor k = $\sqrt{3}$), U_rel_(P) of D-lactic acid was calculated as, $\frac{1-0.99}{0.99\sqrt{3}}\;=\;0.0058$. Correspondingly, the U_rel_(P) of L-lactic acid was, $\frac{1-0.98}{0.98\sqrt{3}}\;=\;0.0118$. U_rel_(M), uncertainty related to standard weighing. The weighed quantities of D-lactic acid and L-lactic acid standards were 55.60 mg and 46.90 mg, respectively. The electronic balance had a verification scale interval (e) of 0.1 mg. According to Chinese Metrology Specification JJG 1036-2022 [31], the maximum permissible error (MPE) is 0.5 e, and the maximum permissible repeatability error is 1.5 e. U_rel_(M) of D-lactic acid was calculated as $\frac{\sqrt{{\left(0.05/\sqrt{3}\right)}^{2}+{(0.15/\sqrt{3})}^{2}}}{55.60}=0.0016$; U_rel_(M) of L-lactic acid was $\frac{\sqrt{{\left(0.05/\sqrt{3}\right)}^{2}+{(0.15/\sqrt{3})}^{2}}}{46.90}=0.0019$. U_rel_(V) is the uncertainty associated with standard solution preparation. Accounting for uncertainties introduced by solution thermal expansion, volumetric flasks, and pipettes, the calculation yields U_rel_(V) = 0.0261. Uncertainty associated with the calibration curve U_rel_(C) = 0.0014 of D-lactic acid, U_rel_(C) = 0.0023 of L-lactic acid, and detailed calculation procedures are provided in Table S4.

For sample assessment, U_rel_(R), the uncertainty associated with repeatability, includes biological variation. Based on precision results from 10 replicate experiments of *L. rhamnosus* CICC 10063R, D-lactic acid and L-lactic acid were calculated separately, resulting in U_rel_(R) = 0.015/1.028/$\sqrt{10}$ = 0.0046, U_rel_(R) = 0.135/15.067/$\sqrt{10}$ = 0.0028. Based on robustness testing results, the U_rel_(CM) calculated for CICC 10063R across different culture media batches were 0.0177 and 0.0070, respectively. The uncertainty arising from the matrix was calculated based on the recovery data obtained from the accuracy results; the U_rel_(ma) values were found to be 0.0086 for D-lactic acid and 0.0099 for L-lactic acid. For both fermentation broth samples and blank samples diluted using a volumetric flask and pipette, the relative standard uncertainty introduced by sample dilution was calculated as U_rel_(Vs) = 0.0117.

In summary, for D-lactic acid, the relative combined standard uncertainty is U_rel_ = 0.0356, and the relative expanded uncertainty is U = 0.0711. For L-lactic acid, the relative combined standard uncertainty is U_rel_ = 0.0335, and the relative expanded uncertainty is U = 0.0670.

### 3.3. Application of the Method in the Detection of Different Strains of LAB

Using the validated method, the 32 strains of LAB were quantified in D-/L-lactic acid, and each sample was analyzed in triplicate (Table 6). These strains were isolated from food products, with some obtained from commercial probiotic products, encompassing 12 genera, including *Bifidobacterium*, *Lactobacillus*, *Leuconostoc*, *Lactococcus*, and so on, totaling 27 species. This collection essentially covers common LAB varieties.

The detection results indicated that strains of the *Bifidobacterium* genus generally do not produce D-lactic acid, with the exception of *Bifidobacterium breve*. Other only L-lactic acid-producing strains included *L. curvatus*, *L. sakei*, *S. thermophilus*, *L. lactis* subsp. *lactis*, *L. cremoris*, and *W. coagulans*. Only D-lactic acid-producing strains included *L. mesenteroides* subsp. *mesenteroides*, *L. mesenteroides* subsp. *cremoris* and *L. delbrueckii* subsp. *bulgaricus*. The highest L-lactic acid producer was *L. paracasei* CICC 24987 at 17.29 mg/mL, while strain *L. delbrueckii* subsp. *bulgaricus* CICC 6047 yielded the highest D-lactic acid concentration of 16.09 mg/mL.

## 4. Discussion

**Figure 5:**
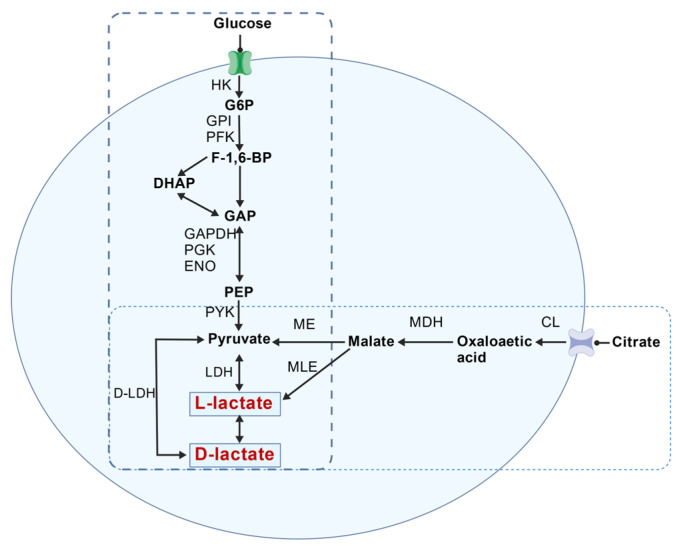
The metabolic pathway of lactic acid.

Although probiotics were often indiscriminately prescribed, they were strain-specific, and different strains may exert profoundly differing effects on the host. While clinical studies should remain the benchmark for the safety evaluation of probiotics, prior to this, in vitro determination of the attributes should be the primary focus for the preliminary characterization of strains. Production of D-lactate by probiotic microbes seems to be an assessment test that needs to be conducted [32]. Probiotic strains require evaluation of D-lactic acid production for safety assessment prior to obtaining GRAS status [33]. Additionally, it was important to note that the ratio of D- to L-lactic acid produced by LAB varies depending on the species and strains. Research demonstrated that the ratio of D- and L-lactic acid produced by the *L. helveticus* strain was 4:3 and 1:1 by *L. plantarum*. In *L. delbrueckii* subsp. *bulgaricus*, more than 90% of the lactate produced through the glycolysis pathway was D-lactic acid [34]. The D/L-lactate ratios detected for the strains in this study were consistent with those reported in the literature. From the perspective of metabolic phenotype characterization, the D/L ratio may serve as a stable characteristic parameter of the strains, providing a reliable basis for distinguishing strains with different metabolic types and for rapidly screening strains with corresponding ratios. As expected, systematic analysis of 32 strains of LAB in this study revealed that different strains exhibit strain-level specificity in lactic acid production capacity, while bacteria of the same species share similar D-/L-lactic acid production ratios. Two isomeric forms of lactate may be formed via reduction in pyruvate by distinct stereospecific NAD-dependent D-LDH or L-LDH, as shown in Figure 5; simultaneously, some LAB possess a metabolic pathway for citric acid that produces L-lactic acid [35].

Previous research found that in fermented milk, *L. delbrueckii* subsp. *bulgaricus* CICC 6047 was a single producer of D-lactic acid, while *S. thermophilus* CICC 6038 was a single producer of L-lactic acid [36], which was consistent with the acid-producing type under the conditions of this study. Moreover, reports found that *S. thermophilus* lacks the enzyme for the D-lactic acid production pathway, while *L. delbrueckii* subsp. *bulgaricus* possesses the relevant enzymes for both D- and L-lactic acid production [37]. Furthermore, investigation had revealed that when the gene encoding D-LDH in *L*. *helveticus* CNRZ32 was inactivated, only L-lactic acid production was detected. In contrast, when the *ldhD* gene was inactivated in *L*. *plantarum*, it still produced equal amounts of L- and D-lactic acid [38]. Therefore, the analysis of genes encoding relevant metabolic pathway enzymes can serve as an important reference, but in practical applications, the detection of strains’ ability to metabolize D-/L-lactic acid and their content is critical and essential.

To our knowledge, enzymatic assays, biosensors, chromatography and mass spectrometry are available for the determination of D- and L-lactic acid [39]. Lacorn [40] validated the performance of the Enzytec^TM^ enzyme reagent kit products for determining D-lactic acid in food and beverages, with the LOQ of 0.015 mg/mL and the maximum measurement range of 0.6 mg/mL, and the relative intermediate precision was between 3.5 and 5.7%. The LOQ was higher than that of the present method, and whether NAD and D-lactate dehydrogenase reagents from different brands have the same or better effects requires ongoing verification. Henry et al. [41] established a method for measuring D- and L-lactic acid in urine by HPLC-MS, which is used for clinical detection of patients with excessive D-lactic acid. It possesses high sensitivity and specificity, combines the high-efficiency separation characteristics of HPLC, and allows for a simplified sample preparation and purification process. In the method developed in this study, samples do not require excessive purification steps, yet achieve specific detection results. Compared to other lactic acid testing approaches, our challenge lay in establishing a stable, accurate, and broadly applicable method, which would facilitate standardization and the implementation of relevant regulations.

The primary consideration was the parameter settings for controlling strain culture conditions, as evidenced by the wide variety of cultivation conditions found in research articles on probiotic safety [32,42]. Additionally, in the GRAS reports of probiotics such as *L. rhamnosus* GG, *L. acidophilus* NCFM, and *B. animalis* subsp. *lactis* Bb-12 [43,44,45], the production of D- and L-lactic acid by the strains was specified. However, the culture conditions are not specified. The levels of metabolites produced by probiotic strains vary depending on nutrient composition and fermentation time [46,47]. Therefore, selecting MRS medium for LAB and defining the cultivation time constitutes a standard and universal strain cultivation method, which serves as a crucial foundation for strain evaluation.

The present study results showed that a recovery extraction was between 93.50% and 105.37%, demonstrating that our simple sample preparation did not cause significant interference from impurity components on the detection results. The study yielded satisfactory results in terms of linearity (R^2^ > 0.9999), repeatability, and reproducibility precision. For the within-run precision (*n* = 10 replicates), the RSD was found to be overall lower than 3.0%, and the inter-laboratory precision (*n* = 6) measured was slightly higher, with RSD no greater than 10.2%; these involve factors such as variations in equipment, operational errors, and differences in strain growth. Minor variations in growth rate and metabolic activity occur during subculturing and cultivation processes, which represent inherent biological heterogeneity. Variations in nitrogen sources, carbon sources, and other components between batches, or minor preparation errors, can affect strain growth and metabolism. Therefore, we had also taken into account the validation of different batches of media. Finally, the short term stability of the sample was evaluated and was deemed to be stable for 2 days stored on refrigerated. Overall, the advantage of this method lies in its ability to be systematically validated and its integration of the entire process from strain cultivation to D- and L-lactic acid detection, making it applicable to various LAB strains. The method had demonstrated stability; its principles were applicable to other matrices, and for entirely new matrices, more verification may be necessary to ensure the accuracy of the results. Moreover, this foundational approach will also pave the way for developing novel methodologies, as exemplified by Augustiniene et al. [48], who developed a transcription factor (TF)-based whole-cell biosensor strategy for the D- and L-lactic acid determination, which was validated by HPLC and enzymatic methods.

## 5. Conclusions

In this study, using an HPLC chiral chromatographic column, we developed a method to simultaneously analyze D- and L-lactic acid produced by LAB, which encompasses three key procedures: strain cultivation, fermentation broth processing, and HPLC quantification procedure. The proposed method was validated in terms of precision, accuracy, specificity, LOQ, linearity, range, and robustness, complying with the performance criteria established by AOAC and ICH for the analysis. Therefore, it was an effective method for strain evaluation, which assesses the content and proportion of D-/L-lactic acid produced by the strain. Using this method, the production of D-lactic acid by various LAB was systematically analyzed. Furthermore, future studies could explore the lactic acid-producing capacity of strains under different environmental conditions, such as in simulated intestinal environments, to enable a more comprehensive evaluation, which will provide a scientific basis for the screening and application of probiotics.

## Figures and Tables

**Table 1:** Information on validation strains.

Number	Latin Name	Strain ID
1	*Lacticaseibacillus rhamnosus*	CICC 10063R
2	*Lactobacillus acidophilus*	CICC 10009R
3	*Bifidobacterium longum* subsp. *longum*	CICC 10068R
4	*Bifidobacterium longum* subsp. *infantis*	CICC 10036R
5	*Lactobacillus delbrueckii* subsp. *bulgaricus*	CICC 6047

**Table 2:** Accuracy for D- and L-lactic acid.

Strain ID	D-Lactic Acid				L-Lactic Acid			
	Spiked Concentration (μg/mL)	Mean Detected Concentration (μg/mL)	Original Concentration (μg/mL)	Recovery%	Spiked Concentration (μg/mL)	Mean Detected Concentration (μg/mL)	Original Concentration (μg/mL)	Recovery%
CICC 10063R	0.00	13.74	13.74	-	0.00	158.94	158.94	-
	20.45	33.45	13.53	97.40	4.16	161.33	157.36	95.44
	61.35	74.52	13.50	99.47	10.40	165.89	156.16	93.50
	102.75	113.94	13.32	97.92	20.80	178.78	158.15	99.21
	143.15	159.64	13.15	102.33	31.20	183.49	152.19	100.34
	184.05	198.73	12.98	100.93	41.60	191.13	150.20	98.38
CICC 10009R	0.00	70.23	70.23	-	0.00	90.43	90.43	-
	20.42	89.58	69.88	96.46	10.40	99.76	89.75	96.23
	40.84	109.57	69.53	98.04	41.60	130.95	88.62	101.75
	71.47	138.13	69.00	96.73	72.80	161.31	87.49	101.39
	91.89	158.25	68.65	97.50	93.60	181.27	86.59	101.15
	122.52	186.85	68.12	96.91	104.00	192.82	86.14	102.58
CICC 10036R	0.00	1.22	1.22	-	0.00	42.58	42.58	-
	10.13	11.01	1.21	96.80	21.45	63.97	42.26	101.21
	50.63	50.83	1.19	98.07	53.63	94.90	41.52	99.55
	101.25	101.02	1.16	98.63	96.53	134.25	40.56	97.06
	151.88	151.21	1.13	98.82	139.43	176.85	39.60	98.44
	192.38	194.32	1.11	100.43	160.88	198.92	38.96	99.43
CICC 10068R	0.00	1.18	1.18	-	0.00	80.08	80.08	-
	10.23	11.85	1.17	104.50	20.80	99.05	79.48	94.08
	51.13	53.05	1.15	101.52	41.60	119.20	78.28	98.38
	92.03	93.40	1.13	100.26	62.40	140.02	77.08	100.87
	143.15	142.77	1.11	98.96	83.20	155.97	75.68	96.50
	194.28	193.33	1.08	98.96	124.80	194.19	73.87	96.40
CICC 6047	NT				0.00	2.45	2.45	-
					41.70	44.57	2.43	101.07
					83.40	86.12	2.40	100.38
					104.25	104.00	2.39	97.47
					156.38	163.09	2.36	102.79
					198.08	203.95	2.34	101.79
CICC 6069	0.00	1.09	1.09	-	0.00	65.77	65.77	-
	10.13	11.62	1.08	104.13	10.73	76.74	65.44	105.37
	60.75	59.60	1.06	96.36	42.90	108.60	64.13	103.66
	111.38	107.14	1.04	95.27	75.08	139.38	62.81	101.99
	162.00	159.14	1.02	97.61	107.25	170.10	61.50	101.26
	192.38	188.05	1.00	97.23	128.70	191.13	60.68	101.37

“-”: not calculated; “NT”: Not Tested.

**Table 3:** Repeatability and reproducibility precision for D- and L-lactic acid.

Strain ID	D-Lactic Acid						L-Lactic Acid					
	Repeatability (*n* = 10)			Reproducibility (*n* = 18)			Repeatability (*n* = 10)			Reproducibility (*n* = 18)		
	Mean Detected Concentration (mg/mL)	SD	RSD%	Mean Detected Concentration (mg/mL)	SD	RSD%	Mean Detected Concentration (mg/mL)	SD	RSD%	Mean Detected Concentration (mg/mL)	SD	RSD%
CICC 10063R	1.03	0.01	1.46	1.10	0.11	10.16	15.07	0.14	0.90	15.41	0.39	2.56
CICC 10009R	7.90	0.21	2.64	7.07	0.52	7.39	8.46	0.17	2.05	8.19	0.63	7.63
CICC 10068R	/	/	/	/	/	/	7.40	0.19	2.61	7.28	0.51	6.99
CICC 10036R	/	/	/	NT			3.72	0.07	1.86	NT		
CICC 6047	16.09	0.26	1.60				/	/	/			
CICC 6069	/	/	/				5.98	0.09	1.49			

“/”: not detected; “NT”: Not Tested.

**Table 4:** Validation results of LOQ.

Component	Spiked Concentration (μg/mL)	Blank Concentration (μg/mL)	Detected Concentration (μg/mL)	Mean (μg/mL)	SD	RSD%	Recovery%
D-lactic acid	0.80	0.60	1.41	1.41	0.04	2.85	101.28
			1.37				95.98
			1.33				91.72
			1.37				96.50
			1.42				102.74
			1.42				103.05
			1.44				105.65
			1.43				104.50
			1.42				102.11
			1.747				108.35
L-lactic acid	0.83	4.37	4.32	4.34	0.02	0.39	94.63
			4.33				96.02
			4.35				97.40
			4.36				99.39
			4.35				97.80
			4.36				99.58
			4.35				97.70
			4.36				99.49
			4.31				93.64
			4.35				97.90

**Table 5:** Validation results of robustness.

Strain ID	D-Lactic Acid						L-Lactic Acid					
	Time (0, 8, 16, 24, 32, 40, 48 h)			Medium (3 Batches, 3 Replicates)			Time (0, 8, 16, 24, 32, 40, 48 h)			Medium (3 Batches, 3 Replicates)		
	Mean Detected Concentration (mg/mL)	SD	RSD%	Mean Detected Concentration (mg/mL)	SD	RSD%	Mean Detected Concentration (mg/mL)	SD	RSD%	Mean Detected Concentration (mg/mL)	SD	RSD%
CICC 10063R	1.02	0.01	1.23	1.10	0.06	5.30	15.09	0.16	1.09	15.09	0.32	2.11
CICC 10009R	8.04	0.12	1.48	7.36	0.39	5.33	8.63	0.16	1.88	8.16	0.17	2.07
CICC 10068R	/	/	/	/	/	/	7.59	0.05	0.63	7.25	0.21	2.92
CICC 10036R	/	/	/	/	/	/	3.77	0.10	2.74	3.64	0.16	4.49
CICC 6047	16.00	0.34	2.11	16.28	0.15	0.92	/	/	/	/	/	/
CICC 6069	/	/	/	/	/	/	5.89	0.27	4.64	6.16	0.18	2.89

“/”: not detected.

**Table 6:** The detection results of D- and L-lactic acid produced by different strains.

Number	Strain	Mean Detected D-Lactic Acid Concentration ± SD (mg/mL)	Mean Detected L-Lactic Acid Concentration ± SD (mg/mL)	Percentage of D-Lactic Acid Content (%)
1	*B. adolescentis* CICC 6070 ^T^	/	7.54 ± 0.13	0
2	*B. animalis* subsp. *animalis* CICC 6250 ^T^	/	2.95 ± 0.12	0
3	*B. animalis* subsp. *lactis* CICC 10067R	/	4.64 ± 0.10	0
4	*B. animalis* subsp. *lactis* CICC 10133R	/	3.70 ± 0.08	0
5	*B. bifidum* CICC 10274R	/	2.79 ± 0.03	0
6	*B. longum* subsp. *infantis* CICC 10168R	/	5.33 ± 0.29	0
7	*B. longum* subsp. *infantis* CICC 10175R	0.03 ± 0.00	6.53 ± 0.51	0.41
8	*B. breve* CICC 10030R	0.06 ± 0.00	2.75 ± 0.22	2.07
9	*L. fermentum* CICC 10053R	6.72 ± 0.16	6.53 ± 0.14	50.72
10	*L. reuteri* CICC 10276R	1.79 ± 0.02	5.83 ± 0.04	23.45
11	*L. helveticus* CICC 10275R	9.62 ± 0.16	6.97 ± 0.11	57.99
12	*L. crispatus* CICC 24879	8.52 ± 0.05	8.06 ± 0.04	51.39
13	*L. delbrueckii* subsp. *lactis* CICC 25164	12.15 ± 0.29	0.37 ± 0.03	97.05
14	*L. gasseri* CICC 24878 ^T^	9.77 ± 0.14	7.11 ± 0.22	57.86
15	*L. johnsonii* CICC 6252 ^T^	9.98 ± 0.30	7.52 ± 0.19	57.04
16	*L. salivarius* CICC 25161	1.04 ± 0.02	15.57 ± 0.33	6.27
17	*L. curvatus* CICC 25172	/	11.31 ± 0.25	0
18	*L. sakei* CICC 6245 ^T^	/	9.83 ± 0.23	0
19	*L. kefiranofaciens* subsp. *kefiranofaciens* CICC 25167	4.48 ± 0.06	1.63 ± 0.11	73.33
20	*L. rhamnosus* CICC 10022R	1.63 ± 0.00	14.90 ± 0.10	9.87
21	*L. rhamnosus* CICC 10029R	1.24 ± 0.02	15.18 ± 0.04	7.54
22	*L. casei* CICC 6117 ^T^	1.03 ± 0.05	15.57 ± 0.33	6.21
23	*L. paracasei* CICC 24987	0.66 ± 0.01	17.29 ± 0.18	3.68
24	*L. plantarum* CICC 25205	8.93 ± 0.03	8.24 ± 0.07	51.98
25	*S. thermophilus* CICC 6038	/	5.12 ± 0.09	0
26	*L. lactis* subsp. *lactis* CICC 6246 ^T^	/	5.38 ± 0.09	0
27	*L. cremoris* CICC 24337 ^T^	/	8.20 ± 0.04	0
28	*L. mesenteroides* subsp. *mesenteroides* CICC 25070 ^T^	6.77 ± 0.25	/	100
29	*L. mesenteroides* subsp. *cremoris* CICC 22181	9.70 ± 0.08	/	100
30	*P. acidilactici* CICC 25166	7.15 ± 0.12	8.24 ± 0.20	46.47
31	*P. pentosaceus* CICC 25165	2.57 ± 0.06	9.74 ± 0.16	20.89
32	*W. coagulans* CICC 25162	/	9.04 ± 0.23	0

“/”: not detected; “T”: type strain.

## Data Availability

The original contributions presented in this study are included in the article/Supplementary Materials. Further inquiries can be directed to the corresponding author.

## Supplementary Materials

The following supporting information can be downloaded at: https://www.mdpi.com/article/10.3390/foods15091537/s1. Table S1: Information of the experimental strains; Table S2: Determination of the calibration curve; Table S3: Verification results of linearity and range; Table S4: Calculation of uncertainty; Table S5: The peak purity match value; Figure S1: Relative residual percentages versus standard solution concentration for two different regression methods of calibration curves: for the ordinary least squares method (red) and the weighted least squares method (blue), with the appropriate weighting factor (1/X^2^) for D/L-lactic acid.

## References

[1] Klotz S., Kaufmann N., Kuenz A., Prüße U. (2016). Biotechnological production of enantiomerically pure d-lactic acid. Appl. Microbiol. Biotechnol..

[2] Kamel K.S., Oh M.S., Halperin M.L. (2020). L-lactic acidosis: Pathophysiology, classification, and causes; emphasis on biochemical and metabolic basis. Kidney Int..

[3] Caldarini M.I., Pons S., D’Agostino D., DePaula J.A., Greco G., Negri G., Ascione A., Bustos D. (1996). Abnormal fecal flora in a patient with short bowel syndrome An in vitro study on effect of pH on D-lactic acid production. Dig. Dis. Sci..

[4] Adeva-Andany M., Lopez-Ojen M., Funcasta-Calderon R., Ameneiros-Rodriguez E., Donapetry-Garcia C., Vila-Altesor M., Rodriguez-Seijas J. (2014). Comprehensive review on lactate metabolism in human health. Mitochondrion.

[5] Ewaschuk J.B., Naylor J.M., Zello G.A. (2005). D-Lactate in Human and Ruminant Metabolism. J. Nutr..

[6] Le A., Cooper C.R., Gouw A.M., Dinavahi R., Maitra A., Deck L.M., Royer R.E., Vander Jagt D.L., Semenza G.L., Dang C.V. (2010). Inhibition of lactate dehydrogenase A induces oxidative stress and inhibits tumor progression. Proc. Natl. Acad. Sci. USA.

[7] Jin S., Chen X., Yang J., Ding J. (2023). Lactate dehydrogenase D is a general dehydrogenase for D-2-hydroxyacids and is associated with D-lactic acidosis. Nat. Commun..

[8] Fabian E., Kramer L., Siebert F., Högenauer C., Raggam R.B., Wenzl H., Krejs G.J. (2017). D-lactic acidosis—Case report and review of the literature. Z. Für Gastroenterol..

[9] Levitt M.D., Levitt D.G. (2020). Quantitative Evaluation of D-Lactate Pathophysiology: New Insights into the Mechanisms Involved and the Many Areas in Need of Further Investigation. Clin. Exp. Gastroenterol..

[10] Remund B., Yilmaz B., Sokollik C. (2023). D-Lactate: Implications for Gastrointestinal Diseases. Children.

[11] Munakata S., Arakawa C., Kohira R., Fujita Y., Fuchigami T., Mugishima H. (2010). A case of D-lactic acid encephalopathy associated with use of probiotics. Brain Dev..

[12] Sheedy J.R., Wettenhall R.E., Scanlon D., Gooley P.R., Lewis D.P., McGregor N., Stapleton D.I., Butt H.L., DE Meirleir K.L. (2009). Increased D-lactic Acid intestinal bacteria in patients with chronic fatigue syndrome. In Vivo.

[13] Rao S.S.C., Rehman A., Yu S., Andino N.M., Andino N.M. (2018). Brain fogginess, gas and bloating: A link between SIBO, probiotics and metabolic acidosis. Clin. Transl. Gastroenterol..

[14] Ku W.H., Lau D., Huen K.F. (2006). Probiotics Provoked D-lactic Acidosis in Short Bowel Syndrome: Case Report and Literature Review. Hong Kong J. Paediatr..

[15] FAO/WHO (2002). Guidelines for the Evaluation of Probiotics in Food.

[16] (2024). Standard for Infant Formula and Formulas for Special Medical Purposes Intended for Infants.

[17] (2023). Standard for Processed Cereal-Based Foods for Infants and Young Children.

[18] (2025). National Food Safety Standard—Safety Evaluation Procedures for Food Microorganisms.

[19] Cevasco G., Piątek A.M., Scapolla C., Thea S. (2011). A simple, sensitive and efficient assay for the determination of D- and L-lactic acid enantiomers in human plasma by high-performance liquid chromatography. J. Chromatogr. A.

[20] National Health Commission of China (2022). Announcement on the Updated “List of Strains Permitted for Use in Foods” and “List of Strains Permitted for Use in Foods for Infants and Young Children”.

[21] Sutula J., Coulthwaite L., Verran J. (2012). Culture media for differential isolation of *Lactobacillus casei* Shirota from oral samples. J. Microbiol. Methods.

[22] Vakili H., Talebpour Z., Haghighi F. (2022). Development, validation, and uncertainty measurement of HPLC-DAD method for determination of some free amino acids in infant formula and medical food products for inborn errors of metabolism. Food Chem..

[23] United States Pharmacopeia (USP) (2017). General Chapter, <1225> Validation of Compendial Procedures. USP-NF.

[24] Pharmacopoeia of the People’s Republic of China (ChP) (2020). General Rule <9101> Guidelines for Validation of Analytical Methods.

[25] ICH (2023). Validation of Analytical Procedures Q2 (R2).

[26] Hoff R.B., Rübensam G., Jank L., Barreto F., Peralba M.C.R., Pizzolato T.M., Díaz-Cruz M.S., Barceló D. (2015). Analytical quality assurance in veterinary drug residue analysis methods: Matrix effects determination and monitoring for sulfonamides analysis. Talanta.

[27] (2008). Uncertainty of measurement Part 3: Guide to the Expression of Uncertainty in Measurement (GUM:1995).

[28] Ellison S.L.R., Williams A. (2012). Quantifying uncertainty in analytical measurement. EURACHEM/CITAC Guide CG 4.

[29] Jang G.W., Choi S.I., Choi S.H., Han X., Men X., Kwon H.Y., Choi Y.E., Lee O.H. (2021). Method validation of 12 kinds of food dye in chewing gums and soft drinks, and evaluation of measurement uncertainty for soft drinks. Food Chem..

[30] Latimer G.W. (2023). Guidelines for Single-Laboratory Validation of Chemical Methods for Dietary Supplements and Botanicals. Official Methods of Analysis of AOAC International.

[31] (2022). Verification Regulation of Electronic Balance.

[32] Mazzantini D., Calvigioni M., Celandroni F., Lupetti A., Ghelardi E. (2022). In vitro assessment of probiotic attributes for strains contained in commercial formulations. Sci. Rep..

[33] Choi I.Y., Kim J., Kim S.H., Ban O.H., Yang J., Park M.K. (2021). Safety Evaluation of *Bifidobacterium breve* IDCC4401 Isolated from Infant Feces for Use as a Commercial Probiotic. J. Microbiol. Biotechnol..

[34] Huang Y., You C., Liu Z. (2017). Cloning of D-lactate dehydrogenase genes of *Lactobacillus delbrueckii* subsp. *bulgaricus* and their roles in D-lactic acid production. 3 Biotech.

[35] Pudlik A.M., Lolkema J.S. (2011). Citrate Uptake in Exchange with Intermediates in the Citrate Metabolic Pathway in *Lactococcus lactis* IL1403. J. Bacteriol..

[36] Ge Y., Yu X., Zhao X., Liu C., Li T., Mu S., Zhang L., Chen Z., Zhang Z., Song Z. (2023). Fermentation characteristics and post-acidification of yogurt by *Streptococcus thermophilus* CICC 6038 and *Lactobacillus delbrueckii* ssp. *bulgaricus* CICC 6047 at optimal inoculum ratio. J. Dairy Sci..

[37] Zhang J., Gong G., Wang X., Zhang H., Tian W. (2015). Positive selection on D-lactate dehydrogenases of *Lactobacillus delbrueckii* subspecies *bulgaricus*. IET Syst. Biol..

[38] Wang X., Zheng Z., Dou P., Qin J., Wang X., Ma C., Tang H., Xu P. (2010). Cloning, expression, purification, and activity assay of proteins related to D-lactic acid formation in *Lactobacillus rhamnosus*. Appl. Microbiol. Biotechnol..

[39] Pohanka M. (2020). D-Lactic Acid as a Metabolite: Toxicology, Diagnosis, and Detection. BioMed Res. Int..

[40] Lacorn M., Hektor T. (2025). Validation of Enzytec^TM^ Liquid D-Lactic Acid for Enzymatic Determination of D-Lactic Acid in Selected Foods and Beverages: Official Method 2024.06 First Action. J. AOAC Int..

[41] Henry H., Marmy C.N., Steenhout P., Béguin A., Boulat O. (2011). Sensitive determination of D-lactic acid and L-lactic acid in urine by high-performance liquid chromatography-tandem mass spectrometry. Biomed. Chromatogr..

[42] Kim T., Mondal S.C., Jeong C.R., Kim S.R., Ban O.H., Jung Y.H., Yang J., Kim S.J. (2022). Safety evaluation of *Lactococcus lactis* IDCC 2301 isolated from homemade cheese. Food Sci. Nutr..

[43] U.S. Food & Drug Administration (2020). GRAS Notice (GRN) No. 865. https://hfpappexternal.fda.gov/scripts/fdcc/index.cfm?set=GRASNotices&id=865.

[44] U.S. Food & Drug Administration (2019). GRAS Notice (GRN) No. 856. https://hfpappexternal.fda.gov/scripts/fdcc/index.cfm?set=GRASNotices&id=856.

[45] U.S. Food & Drug Administration (2019). GRAS Notice (GRN) No. 845. https://hfpappexternal.fda.gov/scripts/fdcc/index.cfm?set=GRASNotices&id=845.

[46] Martín R., Olivares M., Marín M.L., Fernández L., Xaus J., Rodríguez J.M. (2005). Probiotic Potential of 3 *Lactobacilli* Strains Isolated from Breast Milk. J. Hum. Lact..

[47] Farrokh A., Ehsani M.R., Moayednia N. (2018). Lactic acid fermentation of synbiotic cream: Effects on physicochemical characteristics and formation of L (+), and D (–)-Lactic acid isomers. Appl. Ecol. Environ. Res..

[48] Augustiniene E., Jonuskiene I., Kailiuviene J., Mazoniene E., Baltakys K., Malys N. (2023). Application of whole-cell biosensors for analysis and improvement of L- and D-lactic acid fermentation by *Lactobacillus* spp. from the waste of glucose syrup production. Microb. Cell Factories.

